# From chemical metabolism to life: the origin of the genetic coding process

**DOI:** 10.3762/bjoc.13.111

**Published:** 2017-06-12

**Authors:** Antoine Danchin

**Affiliations:** 1 Institute of Cardiometabolism and Nutrition, Hôpital de la Pitié-Salpêtrière, 47 Boulevard de l'Hôpital, 75013, Paris, France

**Keywords:** algorithmic complexity, complementarity, phagocytosis: reticulum, Turing Machine

## Abstract

Looking for origins is so much rooted in ideology that most studies reflect opinions that fail to explore the first realistic scenarios. To be sure, trying to understand the origins of life should be based on what we know of current chemistry in the solar system and beyond. There, amino acids and very small compounds such as carbon dioxide, dihydrogen or dinitrogen and their immediate derivatives are ubiquitous. Surface-based chemical metabolism using these basic chemicals is the most likely beginning in which amino acids, coenzymes and phosphate-based small carbon molecules were built up. Nucleotides, and of course RNAs, must have come to being much later. As a consequence, the key question to account for life is to understand how chemical metabolism that began with amino acids progressively shaped into a coding process involving RNAs. Here I explore the role of building up complementarity rules as the first information-based process that allowed for the genetic code to emerge, after RNAs were substituted to surfaces to carry over the basic metabolic pathways that drive the pursuit of life.

## Introduction

“Man is the measure of all things” (Protagoras), making it difficult to get around an anthropocentric view of the reality that envelops us. Conjectures about the origins of life do not escape this unfortunate shortcoming. Even the quest for our own origin is far from settled: There is no Adam or Eve in the origin of mankind. If you doubt, just try to work out a single-step process that would account for a change from a set of 48 chromosomes (their number in apes) to 46 (their number in man) in a sexed species. Starting with accidental fusion of two chromosomes, a ratchet-like continuum of changes must have distanced us from our ape ancestors. In the same way, it is implausible that there was only one origin of life, as unfortunately many still try to call forth. Thirty years ago, Freeman Dyson provided a convincing demonstration that, contrary to the widespread "adamist" view, which looks for a single origin to all things, there were at least two origins of life [1]. He established that before the emergence of replication processes (making exact copies), a metabolic system must have reproduced (making similar copies), progressively increasing the accuracy of its pathways before allowing a spin-off system to initiate replication. Here I try to pursue this track and go beyond standard views of what life is, and how it emerged, trying to find the simplest ways forward. I focus on one single question, that of the origin of the coding relationship that links the effectors of life functions (in material, molecular terms, the proteins) to the providers of the memory (the genetic program made of nucleic acids) used as a blueprint to propagate them across generations. To this aim, I take the stance of the engineer who, when designing new inventions, tries first to think them in terms of functions. This implies that I combine an abstract view of what life is with its concrete implementation on Earth as we know it. Choosing abstraction first is a way to postpone the restrictions imposed by the intrinsic properties of matter in order to avoid the trite but certainly inaccurate view of life as always made of animal-like creatures.

This presentation entails using the concept of function, a notoriously difficult one [2]. A main problem that lies behind the difficulty of defining what is a function is its relationships with evolution (how did this particular function come into being?), and this is what I discuss. A key idea behind the view I support is that beside the four currencies constituting our world (matter, energy, space and time) we must add a fifth one, information, taken as an authentic physical currency [3]. To make this idea concrete I see cells (and living organisms) as computers, but not those we use today, computers that would be able to generate a progeny of computers [4]. As in common computers, this means a machine and a separate program that is run by the machine. Here, I identify the program driving the life of the cell with its genetic program, chemically embodied in its genome based on nucleic acids and I study how the innards of the machine emerged first. I propose that what we currently know from the analysis of genomes (in particular the functions of the genes that belong to the operating system of life, that we named the "paleome" [5]) gives us hints to progress in our understanding of how life came to being. Finally, among the many functions required for the development of life, the processes that allow aged organisms to construct young ones are of key interest. These processes, in turn, give a direction to the very process of “life and evolution” via accumulation of information, in a ratchet-like manner. Combining “action” with “orientation” will help us to understand the concept of function and how functions keep emerging as life develops.

## Review

### Abstract requirements for the existence of life

#### A fiction

Following “The Black Cloud”, published in 1957 and already based on a very abstract view of life, Fred Hoyle wrote another fictional work for the BBC, “A for Andromeda”, with John Elliot (published in 1962 from the screenplay of a television series [6]). In this book he pictured the remote action, on Earth, of an intelligent civilisation located in the Great Nebula of Andromeda. This action was triggered by an unknown form of life, detected by astronomers as they scanned the universe for non-random signals. A group of British astronomers, in their analysis of the sky –in an effort reminiscent of the still ongoing SETI program [7]– points out an electromagnetic signal within the Andromeda galaxy that does not look random. The scientist who analyses the electromagnetic waves coming from heaven realises that this is not accidental, because the signal is clearly sent in a repeated form by what can only be a scheming intelligence. It takes some time to reconstruct the signal in its entirety because the daily Earth rotation hides it partially. The astronomer then understands that the signal is a message, and that this message has properties reminiscent of a computer program. To decipher its meaning, he runs it as an algorithm in a pioneering computer built thanks to funds from the Ministry of Defence in the mists of northern Scotland. After running first steps of the message in the computer, the astronomer understands that this algorithm is a kind of blueprint for the construction of a new computer. This new computer should combine the calculations run by many small pre-processing computers that must then be introduced into the main frame. The algorithm begins by asking questions about the chemical nature of living matter, and then proposes a scenario for the synthesis of living tissue. The ultimate purpose of the message is to take control of our earthly life.

This fiction is particularly revealing in that it stresses that, while matter is essential in the living objects we see, the key to life is not matter. The entity that is transported from distant stars is physical, yet immaterial (despite photons being its vehicle). It is a piece of information, serving as an invasive and guileful program, not the traditional little green man-like creatures. Life is seen as the physical implementation of a program. In Hoyle's novel, life is the program. An attractive feature of information, vividly prominent in this fiction, is that it is not simply an isolated, worthless independent entity. It may, and must, interact with other sources of information as well as with matter, a feature that someday will need to be included in theories of information. In Hoyle’s novel human action is an intermediary for processing digital messages into material devices. While this touches a key point to understand what life is, it also illustrates a widespread confusion: Because it uses humans as an intermediate, this scenario mixes up the program with its implementation, which requires a specific source of information. Like a virus without a cell to infect, without a living human intermediate, the program would be ineffective, it would not be alive. As in many contemporary views of biology, this fiction is based on an animistic vision, which we might call “the animism of DNA”. This is summarised by the astronomer who discovered the extraterrestrial message: "If we are able to use the computer as a control device, and if we can build a chemical reactor that can act from its instructions as they appear –in fact, if we can make a DNA synthesizer– then I think we can start building live tissue”. Today, it is not difficult to find statements of this kind in connection with the study of the genome of living organisms, and, naturally in scenarios of the origin of life. This is based on the involuntary occultation of what is nevertheless an obvious fact: to run a program requires a machine! We know, certainly, that having a CD with a state-of-the-art operating system (OS) is useless if it is not placed in an actual computer, and that this (information-rich) computer must still be compatible with the OS. Naturally, of course, there is still another feature that is absent in the fiction: creation of a progeny. Yet, this is, as everybody will accept, a core function of life.

### The key role of coding

In parallel with a remarkably prescient vision of cardinal features of life, we find in this book the misunderstandings –the most common ones– of what is today named “synthetic biology” as well as the beliefs spread by mass media, namely the mix-up of a program, the expression of the program, and the machine able to read and express the program (we consistently forget this machine). Just as for superficial minds there are ”genes of” everything (for instance of intelligence, diseases, obesity, and old age), in the novel written by Hoyle, the program is sufficient to establish and produce the final form of the organism whose manufacture it prescribes. It is as if the cooking recipe produced the meal, or rather, a musical score produced the symphony you are listening to. One of the reasons for this deep misunderstanding is that the concept of a program entails a central role for coding, a very deep and abstract concept that is rarely mastered in what is taught in current education systems (the widespread and very misleading use of “genetic code” as a replacement for genetic program is a case in point). The coding process (i.e., using a cypher) establishes a correspondence between the abstract world of information and its material implementation.

**Figure 1:**
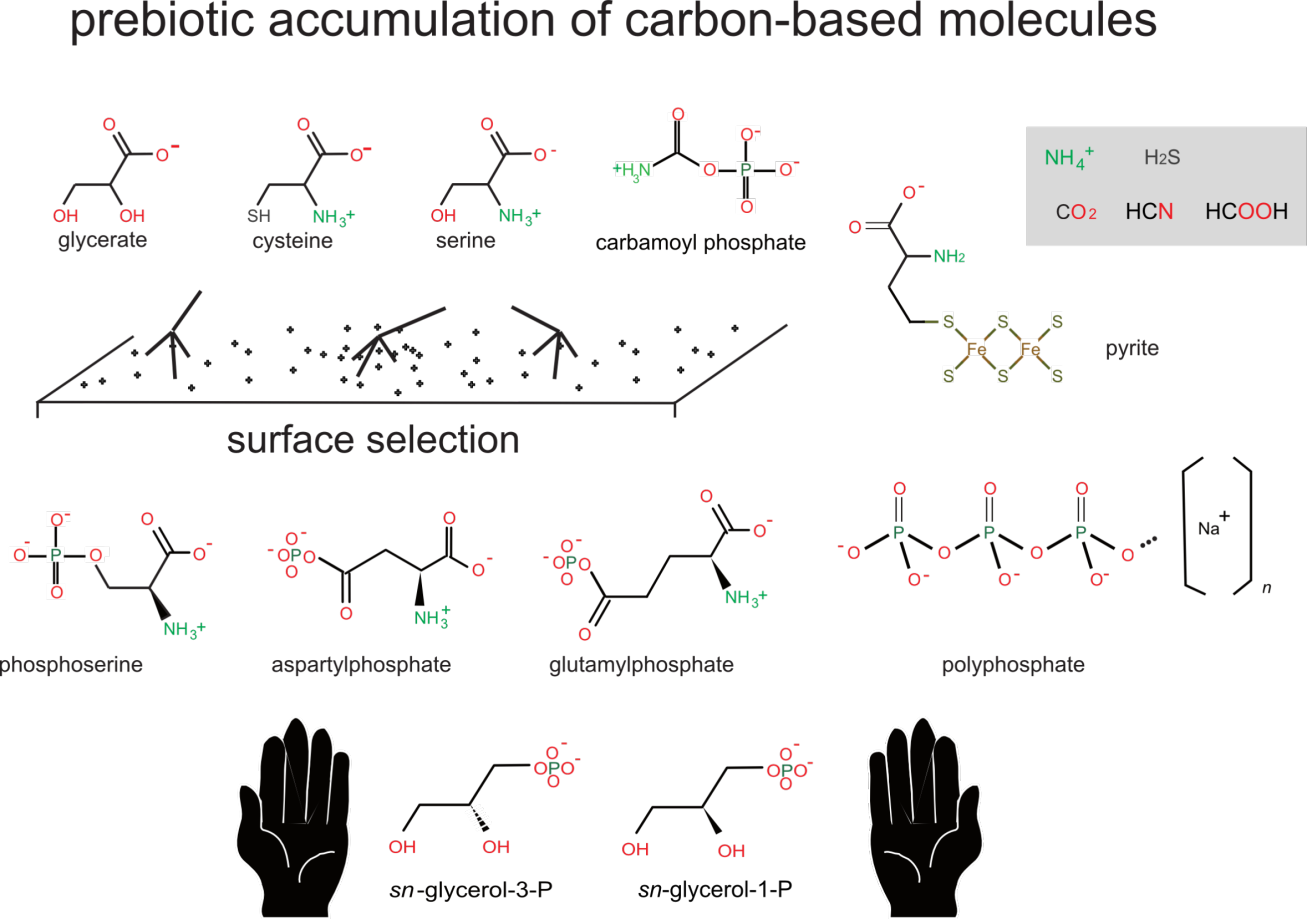
Selective surface metabolism. Prebiotic carbon-based molecules accumulated in a neutral or slightly reducing atmosphere as soon as Earth cooled down. Charged surfaces selectively interacted with charged molecules favouring stereoisomers and reacting in situ to make primary building blocks.

The idea here is that, because our world comprises information as one of its basic currencies, any entity can be described via the use of a symbolic representative, a text written in a finite alphabet (at the most abstract level digitised or, at the very root of coding, represented as a sequence of 0 and 1). The coding process is based on two properties: decomposition of any entity into a finite set of building blocks (amino acids for proteins, atoms for molecules, and protons, neutrons and electrons for atoms) and a correspondence, a code table, between a string of symbols and these building blocks (e.g., for the atomic composition of matter: N for nitrogen, Fe, for iron, C for carbon). Thus a chemical molecule is information-rich. *sn*-Glycerol-3-phosphate can be described, including an outline of its three-dimensional configuration, by a limited alphabet of symbols (e.g., the Simplified Molecular Input Line Entry Specification (SMILES) code table [8]), C([C@H](COP(=O)(O)O)O)O, while its mirror symmetry *sn*-glycerol-1-phosphate is summarised as C([C@@H](COP(=O)(O)O)O)O. That this coding is sufficient (if associated to a concrete machine) is visible in Figure 1, where I used these codes with an algorithm to generate the picture of the corresponding molecules. Remarkably, the link between the genetic program and the effectors of biological processes of the machine that runs the program, the proteins, is mediated by such a code, the genetic code, which establishes a correspondence between the nucleotide building blocks making nucleic acids that carry the program, and the amino acid building blocks making proteins.

This has very deep consequences that ask for a thorough and time-consuming study. The abstract process of coding has given Douglas Hofstadter the subject of a book more than six hundred pages long, “Gödel, Escher, Bach. An Eternal Golden Braid” [9], which despite its depth and length won the popular Pulitzer Prize in 1979. You should never, therefore, expect to understand what life is in two sentences. Even though, after a reasonable effort, you may understand that it is not a large blend of complicated concepts. Instead it comprises just a handful of essential albeit very deep concepts, among which the process of coding has a paramount position. Yet, amusingly, it appears that everybody may talk about biology, give their opinion on natural selection, evolution of the species, or the benefits or misdeeds of genetic engineering. And of course, because this talking does not explore the key questions, it is the most anecdotal characters, accidents and variations that are placed in the limelight, not the profound laws that govern life.

Once more, understanding biology requires a long and deep work, little compatible with the lazy tendencies of the moment. To understand that the key law of life is the coding that relates the memory of the genome to its expression, requires the understanding of the concept and consequences of recursion (i.e., the implementation of a procedure that calls upon itself to determine the subsequent sequence of events), extensively discussed by Hofstadter in his book (again, in some 600 pages). Among its major consequences is an apparently paradoxical property of life: all processes associated to life may be considered as deterministic, but they are not of a mechanical type, as it is, by construction of a recursion, impossible to predict their consequences in the long term (even knowing initial conditions). Living processes are both deterministic and unpredictable. This may read as an oxymoron, but here is a straightforward example using whole numbers (apparently so simple). Knowing the recursive algorithm that allows you to compute the digits of the number π, try to predict the value of the *p*-long sequence (*p* ≥ 1) that follows the *n*-th digit of π (you may associate to this sequence to triggering a major earthquake for example, so that knowing it would matter). Because the only way is to run the algorithm until *n* is reached, this will not be possible if you choose *n* sufficiently large, even with the most powerful computers. Nothing is more deterministic than running this algorithm.

Once this is understood it becomes fairly obvious that cells have abstract properties highly reminiscent of the abstract design of what became our computers, the Turing machine [4]. Indeed this machine combines two separate entities, an authentic machine and a sequence of symbols that acts as a program, controlling the behaviour of the machine. The latter reads, writes and moves the program support (which must be material, but this requirement is not concretely discussed in the abstract formulation of the Turing machine) to reach its symbols. Importantly, exactly as in the living cell, where there is no specific instruction (no design) to tell it to start living, in Turing's description the information manipulated by the machine is purely declarative (i.e., the very presence of the program triggers the running of the machine), and not prescriptive (i.e., there is no need to tell the program to start running). This implies that, for a Turing machine, there is no conceptual split between data and program. Prescription would assume that an external principle prescribes, while there is absolutely no need for any external principle to trigger the onset of life (see the demonstration by Freeman Dyson [1]). Hence, the very word “program” is somewhat misleading. How do we make it concrete, using the building blocks that make cells? And above all, how could a coding process, associating molecules from widely distant chemical classes, proteins and nucleic acids, emerge without some sort of design? A brief scenario for the origin of cells will tell.

### A short scenario for the material implementation of life

Once accepted that life results from the dynamic information processing of organised relationships between material entities, it becomes necessary to identify what those entities are and how they are combined together. Life, as we know it, stems from four well-identified operations: compartmentalisation, metabolism, manipulation and memorisation. The former two operations are performed mainly by small molecules (carbon-based and comprising a few tens of atoms), whereas the latter two are carried out by macromolecules (nucleic acids and proteins, made of a limited number of building blocks). To these operations we must add two essential laws, complementarity and its major consequence, coding (just brought up as key to life).

### Making cells

#### Compartmentalisation

The atom of life is the cell, and a cell generate cells: “omnis cellula e cellula” [10]. The obvious function associated to this view is that the cell separates between an inside and an outside. In 1935, James Danielli proposed with Hugh Davson that this embodiment was achieved by formation of a bilayer made of amphiphilic lipid molecules [11]. This process is entropy-driven (life belongs to physics, it is not a fight against the second principle of thermodynamics), using the global distribution of water molecules as a driving force to order lipids into cell-like structures (of a considerable variety, even in bacteria [12]). Membranes also contain proteins as essential components. It took very long to understand the way proteins interact with membrane lipids, and our knowledge in the domain is still far from complete. There exist many models describing the operation (including ideas about the asymmetry of the bilayer, its local changes and lipid rafts). Work exploring the way proteins are inserted into membranes is a thriving domain of research [13].

Membranes serve a variety of functions such as transport, sensing, protection or supporting movement. They are also involved in energy production via vectorial transport of ions, generally protons. Transport and management of energy imply manipulation of the electro-chemical gradient built up between the inside and the outside of the cell (in particular with the fascinating nanomotor ATP synthase [14]). Membrane components age and waste away: This implies maintenance. Finally, there are specific needs to allow for division while the role of the membrane differs during states of growth and non-growth. The former implies a constructive function of the membrane. Proteins are the effectors of this function with the key operation of allowing protein insertion within the membrane.

Studies investigating spontaneous evolution of lipid vesicles showed that they split, fuse, get internalised and make complex internal networks [15]. Beside lipids, polypeptides form coacervates, which also allow for compartmentalisation [16]. A main difficulty to understand the process is that membrane proteins must fold within two-dimensional (2D) bilayers. This implies the management of construction and maintenance within a 2D structure, while the metabolism that develops in the cytoplasm and produces the building blocks for membranes and their proteins is expressed in three dimensions (3D). Matching the syntheses in both compartments is not a trivial matter because adequate tuning of the corresponding rates of synthesis depends on the volume that will be occupied by the synthesised compounds. Remarkably, rather than in prokaryotes, this hurdle is much easier to solve in eukaryotes with their endoplasmic reticulum, which is a kind of membrane structure folded within the cytoplasm as a Peano surface, thus solving the 2D/3D dilemma. It is therefore natural to assume that the first cells harboured an internal membrane network [17] coupled to peptide metabolism.

Finally, an essential feature of compartmentalisation is more subtle: A cell must give birth to another cell. This implies that its envelope is susceptible to growth and division. In summary, this early key function to life is inseparable from the existence of proteins, or, at least of chemical compounds related to proteins.

#### Metabolism

Life is not static. Dormancy, that we find in the microbial spore or the plant seed, is an intermediary state between life and death. But it will only be associated to life when an organised set of dynamic processes, metabolism, starts to unfold. As its Greek name implies metabolism is a (chemical) state of flux. It drives the construction of molecules from smaller parts (anabolism) and the breakdown of the larger ones into smaller parts (catabolism), building up the individual components of the living machine, and the energy needed to run it. Metabolism follows a logic that accounts for the reason why a narrow subset of atoms and molecules has been retained [18]. To make a long story short, the atoms of life must both be abundant in the universe and form stable covalent bonds at 300 K in water. In order to carry as much information as possible the building blocks of life must be able to polymerise and form macromolecules. Again, this can be driven in water by an entropy increase, if a selection process retains the macromolecules in a specific compartment. Surface metabolism at the origin of life is perhaps the simplest way to harness this ubiquitous property of thermodynamics. Samuel Granick, very early on, remarked the important role of transition metals in biological processes. He further noticed that extant metabolism was organised around common minerals on which biosynthetic chains developed extending his view to an experimental approach [19]. Later on Wächtershäuser refined this view and proposed that iron–sulfur centres were the organising minerals [20]. Despite some efforts, we still lack experiments, however, that would trigger a convincing scenario for a mineral origin of metabolism. This lack of experimental substantiation may be due to the fact that our present reflection on surface metabolism is driven by carbon chemistry, while the question of nitrogen availability, as discussed below, may be a central limitation to prebiotic scenarios. As a chemical constraint that must be accounted for, the building blocks of proteins, amino acids, do not make a random collection at all. A subset is found repeatedly in outer space (e.g., glycine, the smallest amino acid is even found in comets [21], and meteorites contain alanine and aspartate as well as many other common proteinogenic amino acids [22]).

Many other scenarios for prebiotic chemistry have been proposed. Most rest on the popular view of a prebiotic soup, which allows for the use of active gaseous molecules such as HCN or H_2_S, further activated by UV light [23]. Continuous synthesis of ribose would be a difficult challenge to solve, and first studies described a possible scenario with arabinose aminooxazoline instead. A solution for the synthesis of ribose aminooxazoline was recently proposed by the same author and his colleagues [24]. However, while these syntheses may operate under relatively mild conditions with compounds from volcanic emanations, they still need to be complemented by an entropy-driven process favouring polymerisation. Alternating dry and wet episodes might provide an efficacious mechanism, but this involves surfaces in a straightforward way. Furthermore, it is still essential to associate prebiotic processes with selective steps that would retain only compounds that will evolve further into biomaterials. Surfaces, again, are a natural way forward. In summary, the most likely compounds that make the very first metabolic pathways are charged compounds with one to three carbon atoms, amino acids and a variety of peptides or related compounds, certainly not RNA [25].

Phosphates, with their remarkable metastable state in water were selected as surface attachment groups and first units involved in energy exchanges [26]. Alternating drying steps followed by rains or floods resulted in the condensation of phosphate moieties on many primeval compounds. These include serine as serine phosphate and aspartate protected against cyclisation as aspartyl phosphate (Figure 1). This created a collection of charged metabolites that would stick to surfaces and come in contact with each other, promoting a variety of reactions. The first stages of reproductive surface metabolism were prone to produce charged variants of peptides. Among the minerals that would carry over the first (iso)peptide-based metabolic pathways one finds iron–sulfur clusters (pyrite) [20] and polyphosphates [27]. Obviously, selected peptides would be part of the first prebiotic building blocks and compounds, exhibiting a range of promiscuous catalytic activities. This includes hydrolytic self-degradation (proteolysis). Interestingly, rather than working against the ubiquitous presence of polypeptides during early steps of metabolism, this activity opened up a complementary function, that of resisting proteolysis. This created an essential selective step that enriched metabolic pathways with a limited subset of stable active peptides and derived compounds. Finally, surface selection is prone to favour specific spatial shapes. Symmetry is an unstable condition with symmetry breaking the rule [28]. It had to be broken in the choice of amino acids for building polymers, exactly as we have to drive either on the right or on the left to prevent collisions or traffic jams. Any accidental local enrichment of a particular shape would be symmetry-breaking. This contingent pick is a straightforward explanation of the ubiquitous presence of one family of stereoisomers, L-amino acids, in proteins.

**Figure 2:**
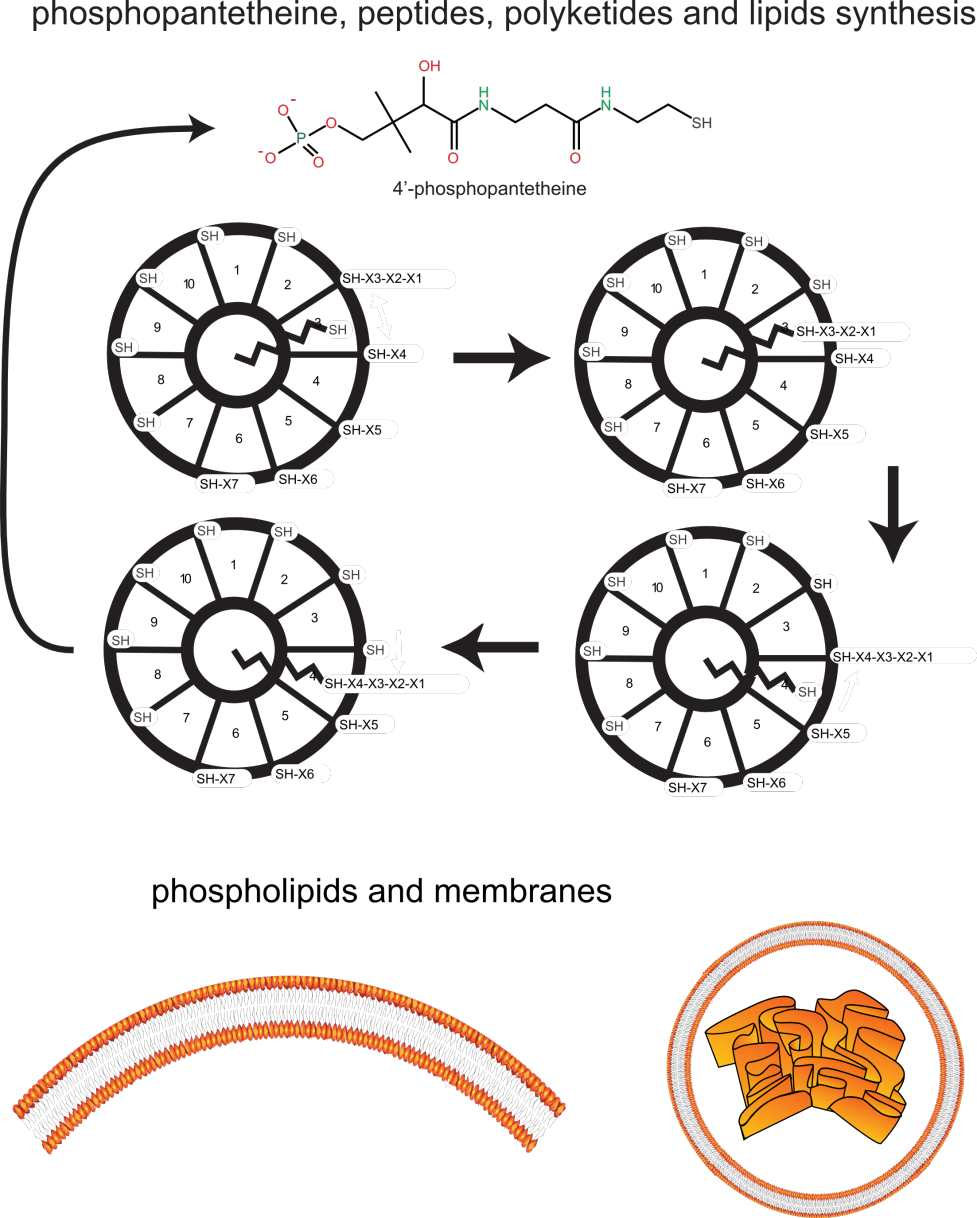
Building up membranes, peptides and co-enzymes. Thioester-based metabolism resulted in the synthesis of a variety of precursors of coenzymes (including 4′-phosphopantetheine as an isopeptide), lipids and peptides, via a swinging-arm catalytic engine.

Remarkably, most coenzymes –necessary effectors of metabolism, the existence of which is a prerequisite for any plausible scenario of origin– are today synthesised from simple carbon molecules and amino acids. Among those, 4′-phosphopantetheine (cysteine condensed with pantothenate, a derivative of valine synthesis, and a phosphate as a charged group) has the remarkable role of a swinging arm transporting a variety of thioester substrates between sulfhydryl catalytic sites (Figure 2). It could well have been involved in its own synthesis as well as that of diverse compounds involving acyl groups (lipids, essential for compartmentalisation [29]), a variety of (iso)peptides as in the synthesis of fatty acids today, non-ribosomal peptides and polyketides [30]. The involvement of thioesters in a primitive metabolism, predating the systematic input of phosphate has been documented by Segré and co-workers in a convincing way [31]. Other coenzymes, possibly generated by such a swinging-arm thioester-dependent catalysis, may have been precursors of nucleotides, the essential building blocks of nucleic acids. As a matter of fact, extant biosynthesis of nucleotides (built on purine and pyrimidine carbon–nitrogen aromatic heterocycles) is based on the incorporation of amino acids in the core of nucleotide precursors. Pyrimidine nucleotide biosynthesis uses aspartate and combines together ubiquitous molecules, water, carbon dioxide, ammonium and phosphate (forming carbamoyl phosphate, also a precursor of arginine, an amino acid absent from the very first steps of prebiotic metabolism), while purine biosynthesis combines glycine and aspartate, together with phosphorylated derivatives of ribose.

These pathways open up a major chemical challenge. Ribose is a very unstable metabolite. Any scenario that advances nucleotides (and even more RNA) at the origin of life should be able to account for a steady synthesis of this molecule. In passing, this also argues fairly strongly against an origin involving hot temperatures, because heat considerably increases ribose instability [32]. Another argument for a late appearance of ribose is the following: Sugars involved in anabolism are essentially of the D-isomer type. This results from selective evolution involving competition with L-amino acids in early essential processes [33]. As a consequence, we can be fairly confident that ribose, and therefore nucleotides, appeared after an (iso)peptide-based metabolism was commonplace.

**Figure 3:**
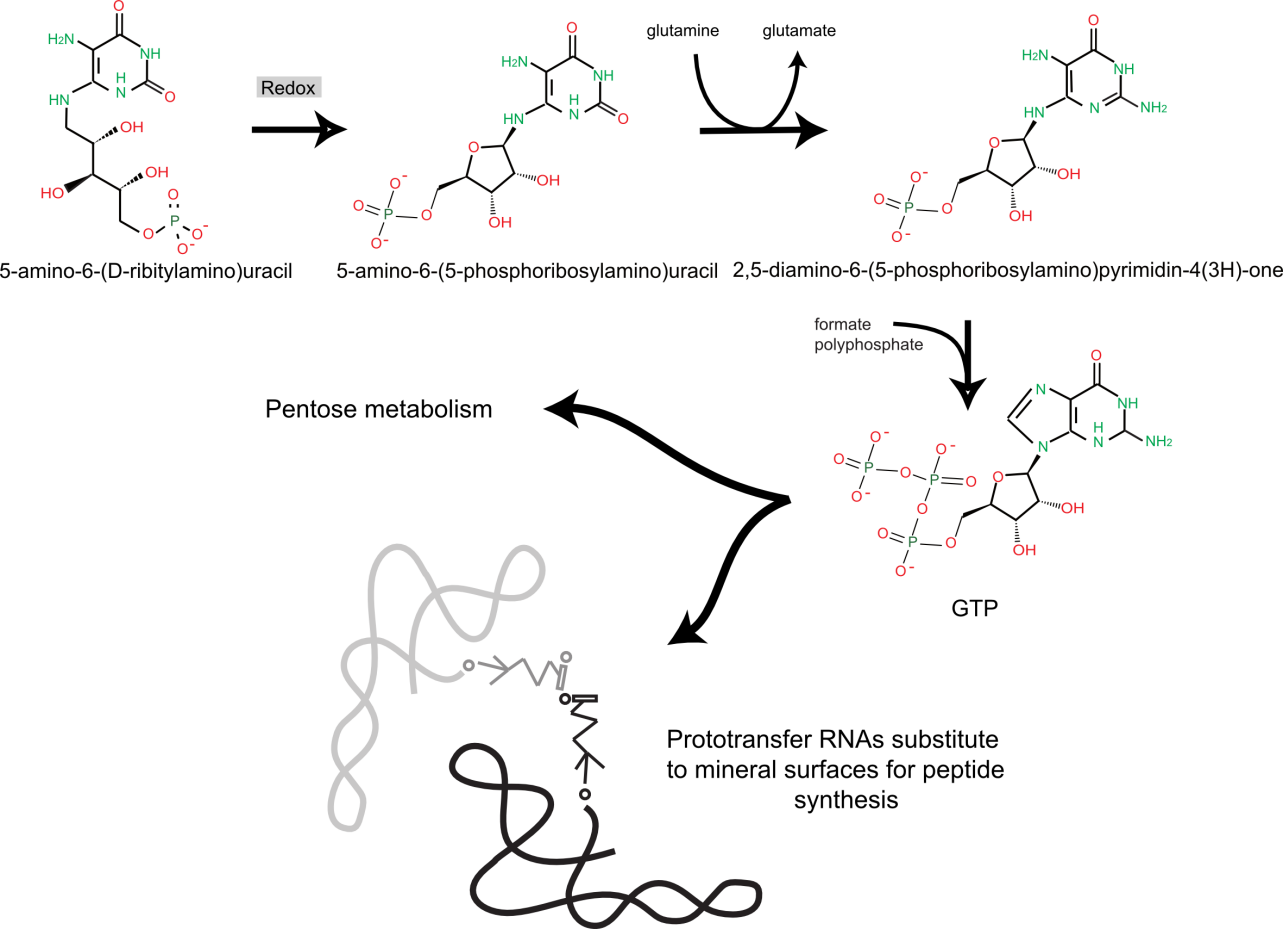
The RNA metabolism world. Among molecules built up by a swinging-arm thioester are pyrimidines coupled to reduced phosphocarbohydrates. This may lead to direct synthesis of nucleotides and later RNA metabolism coupled to ribose metabolism.

Cofactors such as pterins and riboflavin are ubiquitously present in living organisms. Precursors of these essential compounds may have been synthesised by a thioester swinging-arm pathway and phosphorylated by polyphosphate. Remarkably, in living cells these pathways associate interconversions between purines and pyrimidines [34–35]. Furthermore, the nitrogen-rich intermediate 5-amino-6-(D-ribitylamino)uracil comprises building blocks that are commonly found under prebiotic conditions. Once phosphorylated (as discussed previously, via alternation of dry and wet conditions), this molecule would be reduced to a compound containing a 5-phosphoribosylamino group. Simple steps would finally condense formate in the presence of pyrophosphate, leading to phosphorylated guanosine, without requiring a prior synthesis of ribose. The only specific requirement would be that some catalysis allowed for a redox reaction (this is a general requirement of cell metabolism, involved in many metabolic steps, that is difficult, if not impossible, to fulfil using only RNA). As a consequence, primitive metabolic pathways would subsequently synthesise general precursors of nucleotides via phosphorolysis, allowing for both the synthesis of all nucleotides and the creation of a carbon metabolism derived from D-ribose-phosphate (Figure 3). This scenario is of course a bold conjecture but it illustrates how syntheses based on the activity of thioesters [36] and a surface-bound swinging arm may have produced a variety of metabolites.

#### Manipulation

In contrast to metabolism and compartmentalisation, manipulation and memorisation involve entities that are not small molecules but molecules made of thousands, millions, sometimes billions of atoms. These processes organise and rule the flow of information that is key to life. The synthesis of macromolecules requires an abundant supply of basic building blocks produced by metabolism. Up to this point, we have followed Dyson’s reasoning. We have assumed that small-molecule metabolism progressively improved autocatalytic cycles producing and retaining a limited dictionary of building blocks enclosed in lipid vesicles made semi-permeable by peptides. These chemical reactions required functional catalytic power to handle substrates and reject products for further manipulation. The swinging-arm conjecture is a telling illustration of the way peptides and, later, proteins may be proficient in creating metabolic functions.

In order to fulfil their main functions, exploration of the environment and generation of a young progeny, living cells must display a huge variety of further actions that allow for the construction of cells, as well as transport across membranes, movement for exploration (including predation), protection against accidents and management of competition, but also repair, sensing and regulation. Almost all of these actions are operated by proteins in extant living organisms. A major question, therefore, was the understanding of the processes that made them come into being. Scenarios for the emergence of catalytic properties have been briefly outlined above as synthesis of peptides and coenzymes. Information management will be tackled later on when we consider the laws of complementarity and coding.

#### Memorisation

We now need to consider the process of memorisation that allows primitive cells to transmit to their progeny some of the information they collected as metabolism proceeded and evolved. A living organism is autonomous. To develop and survive it rests on the existence of some entity that is propagated from a generation to the subsequent one, a blueprint, a memory. This memory will perpetuate, as exactly as possible, the information that controls the birth and development of the organism, from generation to generation. An early level of memorisation is present in autocatalytic metabolic cycles, but, as noticed by Dyson, this is an unstable way to keep traces of past events [1]. A further memorisation step, at the origin of replication, must have followed the reproduction of metabolism. Concretely, in living cells the replicated substance of the blueprint memory of the cell is its genome, which is made of nucleic acids. Let us be guided by Dyson again and remark that, because this step is considerably more accurate than the fairly fuzzy reproduction of metabolic pathways, it must follow, not predate, the time when protein-based processes (the manipulation stage) emerged. In line with the Andromeda metaphor, in cells, the memory heritage is made of the chaining of nucleotides, summarised as a sequence of letters, similar to the words and sentences of an alphabetical text.

These processes, memorisation and manipulation, are tightly linked to two fundamental information managing laws, complementarity, accounting for the vertical transmission of memory, and coding, allowing for the correspondence between the carriers of the processes of memorisation and manipulation.

### Managing information

#### Complementarity

In biology, complementarity is a feature of reality based on asymmetric shapes of molecules [37]. After discovering that only one 3D form of tartaric acid was present in the lees of wine Pasteur claimed “La dissymétrie, c’est la vie” (dissymmetry, this is life). Indeed, the carbon-based molecules of life are restricted to a subset of compounds with identical chemical structure but diverse 3D structures, selecting only a very limited panel of stereoisomers among those possible (for example, there are four isomers of the amino acid isoleucine, but only L-isoleucine is proteinogenic, while D-isoleucine and L- and D-*allo*-isoleucine are not). This ubiquitous dissymmetry is the basic level on which life manages information [38]. Asymmetry has an important consequence: It creates a set of highly stereospecific environments, leaving room for a particular complement, as described by Fisher in his lock-and-key image of enzyme catalysis [39], or in the widespread image of the antigen–antibody interactions during the immune response [40]. Complementarity illustrates a formal correspondence that may be used subsequently as a recognition signal. It manages information as signals in sensor–receptor interactions. This is exploited in living organisms in the way sensors monitor their environment. For example, there are receptors for taste with exquisite recognition of specific molecules, sugars for example. The sweet taste is triggered by a lock-and-key process in which sugars fit within a specific cavity of the receptor. This interaction can be mimicked by compounds that have nothing in common with sugars or with each other, such as the highly ”sweet” but completely unrelated proteins thaumatin and monellin [41]. Within cells, networks of protein interactions are mediated by rules following complementarity patterns that are yet to be discovered, but are central for the genetic or epigenetic build-up of functions after selective stabilisation [42].

A noteworthy case of the complementarity rule, possibly protein-related and associated to a duplication process, is widespread in eukaryotes. These cells consistently possess protein structures based on tubulin subunits, the centrioles, which undergo exact duplication in each generation. The process that drives this duplication of a protein structure is still a matter of speculation [43]. Centrioles are cylindrical protein complexes with a nine-fold symmetry that is broken with a very precise timing when cells prepare to produce a progeny. Following this symmetry-breaking event of yet unknown origin, a set of priming proteins attaches at a specific site to the outside of the parent centriole. It then progressively builds up, orthogonal to it, a pre-centriole which, once completed, will separate from the parent as a full blown centriole. This daughter organelle will then play the same role as that of the parent for organising chromosome distribution in the daughter cell. This structure is remarkable as it is apparently a protein-only structure that undergoes exact duplication. However, the parent structure is not used as a template for the daughter, as in nucleic acids, for example. In fact, the entity that is replicated is not a protein complex but an algorithm of construction. Hence, in this particular instance, replication is not a protein-replication system, nor is it directly associated to nucleic acids used as templates. The algorithm that drives duplication of the centriole is a piece of information with delayed implementation, associated to a specific set of genes that are replicated when the cell reproduces.

Protein-network replication might have predated replication mediated by nucleic acids, via organisation of information mediated by the formation of protein complexes. However, direct peptide replication has not been observed in biology yet, although it has been demonstrated in artificial systems [44–45]. Complementarity is ubiquitous in protein interactions but varies extremely. The situation where complementarity is the most obvious feature of processes of life is that of interactions involving nucleic acids. In these molecules, complementarity, which leads to the famous double helical structure, is a straightforward consequence of steric rules between isosteric piles of pairs of purines and pyrimidines. This opened up the idea that a primitive coding process was at work during replication, with one strand of DNA entirely specifying the complementary strand. However, this first rule does not solve the riddle of the correspondence between the sequence of DNA and that of proteins, which requires a higher level of coding.

#### Coding

Coding is a case of organised complementarity used in a repetitive way. Because of its intrinsic asymmetry, any biological form creates, by default, the possibility for complementary interactions, opening up a recognition process similar to that using a code. A remarkable consequence is that this is an abstract way to create an association between matter and information, exactly as the integer “3” can be coded in a variety of languages (e.g., three, trois, τρια, 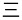). The one-to-one correspondence of complementary strands in nucleic acids was a straightforward coding process, but suggested that there could be a coding rule associating the DNA sequence with that of proteins in which amino acids are chained exactly as nucleotides are chained in nucleic acids.

In an astute analysis of the double-helix structure of DNA, George Gamow remarked that the possible diamond-shaped pockets in the 3D grooves of the double helix were of 20 different types, exactly matching the number of proteinogenic amino acids. Each pocket is defined by specific arrangements of the four nucleotide bases. This “diamond code” is made of 20 overlapping triplets suggesting that each amino acid in the corresponding polypeptide sequence is determined by a group of three bases in the corresponding section of the nucleic-acid chain [46]. However, the overlap between the sides of the consecutive pockets imposed an overlap in the corresponding coding nucleotides, telling that some sequences of amino acids should never be observed in proteins of biological origin. Yet, proteins in data libraries displayed such “impossible” sequences. This demonstrated that while one needs at least three bases to encode 20 amino acids (doublets of four nucleotides would code at most for 16 amino acids), the code is unlikely to be overlapping.

Later on, two major discoveries changed the picture and established the modern way to see how proteins are translated from their gene. It was found that the process required two code-dependent “rewriting” steps, a first step using the minimal one-to-one complementarity code between DNA and RNA nucleotides (transcription), followed by a machinery that operates in a way quite similar to that reading the tape of the Turing machine (translation). Nanomachines, the ribosomes, read contiguous (not overlapping) triplets of nucleotides (codons) in succession. To this aim they use specific transfer RNAs (tRNAs) loaded with amino acids, which use a possibly degenerate RNA complementary code (using the triplets that form anticodons) to establish the correspondence between the codons and each of the 20 amino acids.

Some triplets (for example, the four codons ACN code for threonine) are ambiguous, imposing that they are sometimes deciphered by different tRNAs. The consequence is that there are always more specific tRNAs than amino acids [47], although less than the 61 codons specifying the amino acids (three codons are used to mark the end of the gene coding region that has to be translated into a polypeptide) because the codon–anticodon interaction can use a relaxed complementarity rule. This situation led to a further coding requirement between a specific tRNA and its cognate amino acid (anticodon–amino acid correspondence). As in the case of complementarity in protein complexes there is no general rule establishing this correspondence. It is more or less ad hoc, obviously the result of historical events that governed the origin of the translation process [48]. Perhaps, if we follow a reasoning similar to that of Gamow, it emerged via direct interaction between each amino acid and a cognate anticodon [49].

This observation establishes that a more or less contingent sequence of events is at the origin of the way the genetic code emerged. It was based on the concomitant presence of amino acids and RNAs elicited by the local constraints of chemistry and geology. This simultaneity channelled information into the formation of the first living organisms.

#### From substrates to templates, RNAs at the origin of the genetic code

Among the many codes still to be discovered, the genetic code is at the heart of life. Having set the stage, we now can try to understand how such an abstract operation as that of the correspondence between the sequence of DNA and that of proteins could have come to being. The most straightforward process would have been a direct interaction between amino acids and nucleic acids, as imagined by George Gamow. However, in the absence of any design, things could not develop in this intelligent way, but unfolded more slowly. The actual emergence of the genetic code required a succession of small steps involving progressive improvement of peptide-based metabolism. As expected from a stepwise development, this process created a fair number of anecdotal features that were consequences of purely historical events. This clarifies why the implementation of general abstract laws, such as those driving recursive gene expression, was systematically plagued with “illogical” (for the planning mind of an engineer) tracks, making biology fairly difficult to grasp for the unprepared mind.

#### The RNA-metabolism world

The reproduction of progressively more efficient metabolic pathways preceded the replication of nucleic acids (perhaps in parallel with the replication of proteins, as we saw with the centriole example). A key question is now to understand how both processes could be linked together, associating proteins and nucleic acids. What we discussed above can be summarised with the words of Monnard: “(1) The synthesis of RNA monomers is difficult; (2) efficient pathways for monomer polymerization into functional RNAs and their subsequent, sequence-specific replication remain elusive; and (3) the evolution of the RNA function towards cellular metabolism in isolation is questionable in view of the chemical mixtures expected on the early Earth” [50]. We have left our scenario of the origins of the first cells at a moment when peptides and nucleotides were present simultaneously in cell structures likely to associate an outside envelope and a variety of internal membranes supporting metabolism. Subjected to an alternation of dry and wet conditions ribonucleotides began to polymerise [51]. However, if not associated with other molecules, this polymerisation involved both free hydroxyl groups of ribose, resulting in a mixture of 2′,5′- and 3′,5′-phosphodiester bonds. By contrast, when peptides are present in the mixture, polymerisation is essentially happens through 3′,5′-phosphodiester bonds [52], stressing again the importance of peptides at the onset of prebiotic nucleic-acid chemistry.

At this point, RNA molecules with 3′,5′-bonds were formed. They are flexible molecules that explore the formation of double-stranded regions based on a relaxed complementarity code (A–U and G–C or G–U), forming stems and loops. This situation has long been investigated [53]. It is the basis of a considerable number of works about RNAs involved in a large number of functions, including catalytic activities (ribozymes). It can therefore be expected that primeval metabolism was developed at this point as a mixture of peptide- and RNA-mediated catalytic activities, within protocells. Because of their structures, RNA molecules could easily become substrates for metabolic reactions [54], progressively substituting the mineral surfaces that had been present at the onset of metabolism [34]. This defined the stage of the RNA-metabolism world.

Notably, the involvement of these RNA molecules in pre-translation metabolic processes is still prominent in a variety of metabolic reactions where tRNA molecules are definitely uncalled-for. This is the case for the pathway to an essential cofactor of electron transfers, heme (synthesis of aminolevulinate [55]), and above all, of membrane components such as aminoacyl phospholipids [56]. This is also consistent with the observation that some non-ribosomal syntheses of (iso)peptides are performed by enzymes highly related to class-I transfer RNA synthetases [57], in keeping with a simultaneous development of non-ribosomal protein synthesis and RNAs. Furthermore a variety of activated aminoacyl tRNAs are modified by homeotopic (or pre-translational) modification [34,58], reminiscent of what could be a role of tRNA as support of group transfer in early metabolic pathways. This includes asparaginyl, glutaminyl and selenocysteyl tRNA, as well as formylated methionyl tRNA for the initiation of translation.

All these observations can be considered as archives of past metabolism [54,59], with tRNA ancestors as key support molecules. Interestingly these processes must have started with molecules shorter than the ca. 76 nucleotide-long extant tRNA, which still display a variety of forms [60]. As a case in point, Hopfield remarked that tRNAs are probably the result of an early duplication, and that they could have been involved in the selection of amino acids interacting with the region that now forms the anticodon loop [49]. An interesting time line for the origin and evolution of tRNA has been proposed recently [61]. With ribozymes involved in the catalysis of peptide-bond formation, and primal tRNAs as handles carrying amino acids used in the process, an alternative or complement to the swinging-arm peptide synthesis would have evolved in parallel, with RNA-dependent peptide synthesis progressively taking the lead. At this point RNA molecules are substrates involved in metabolic pathways and in catalysis. In parallel, the complementarity law allowed for fuzzy pairing between RNA molecules (in particular G could pair with U in addition to pair with C). Ongoing polymerisation of ribonucleotides resulted in the emergence of a new function. Polypeptide synthesis used RNA substrates carrying amino acids and RNA ribozymes (the forerunner of the ribosomal RNA peptide centre) for peptide formation. Subsequently, another class of RNA molecules complementary to part of the tRNA ancestors carrying amino acids created a positive interference in this process that improved the formation of peptides. This class of RNAs behaved as templates to order the amino acid residues of the peptides into a well-defined sequence.

#### The RNA-genome world

Accumulation of these latter “peptide sequence-specifying” templates of RNA sequences matching the peptide sequence via a coding process, asked for the synthesis of their exact copies, i.e., replication. This operation evolved from natural RNA catalytic activity [62] and progressively improved its autocatalytic reproduction by using increasingly more accurate complementarity rules (i.e., limiting the fuzzy complementarity rule used in specific peptide synthesis to standard A–U and G–C pairs). While this would perhaps also have been possible in a pure RNA environment, it was assisted by the same class of co-evolving molecules, the peptides that had favoured the formation of 3′,5′ over 2′,5′ bonds. Furthermore, peptides were also necessary for the machinery to help separating replicated strands, allowing for a further round of replication [63]. It can therefore be expected that RNA replicases evolved rapidly, in parallel with non-RNA-directed peptide synthesis.

In summary, these primitive enzymes associated an RNA molecule capable of catalysing peptide-bond formation (the ancestor of the ribosomal RNA peptidyl transferase centre) and the resulting protein functioning as an RNA-dependent RNA polymerase [64]. Today, and this is further evidence of early roles of tRNAs in RNA metabolism, viral RNA replicases still initiate replication using tRNA-like structure as primers, involving these molecules in yet another non translation-related function. Together with the previously discussed view of ancestor tRNAs as handles carrying over metabolic pathways, this supports the idea that these structures are archives of past RNA replication processes [65]. These replicases had to evolve in parallel with the synthesis and replication of ribosomal RNA. A variety of models involving ribozymes and introns of the group-1 family have been proposed to account for this parallel requirement [66]. This view of the RNA-genome world summarises in fact the widespread accepted view of the RNA world, which, in the absence of the idea of an RNA-metabolism world, obscures all the metabolic steps that would have been necessary for stable synthesis of the nucleotides essential for building up RNA [67].

#### The first cells and their descent

In the same way as coacervates can multiply compartments within a single entity [16], phospholipid vesicles form a variety of cell structures, involving vesicle engulfment [17]. It is therefore quite plausible that the RNA-metabolism and RNA-genome worlds were combined together within a single cellular entity, replete with membrane structures (Figure 1), that displayed a general tendency for a primitive form of phagocytosis [68]. While it is routine to think that smaller means less complex and more primitive, comparing the huge Electronic Numerical Integrator And Computer (ENIAC, 1945) with your cell phone tells you that this is a widely mistaken assumption. The saving of space, matter and energy tends to evolve toward miniaturisation, and highly evolved forms are often much smaller than their ancestors. This makes it likely that these primitive multicompartment cells were considerably bigger than most of the extant bacterial cells (although the variety of forms and sizes they can display is huge [69]), which are certainly highly evolved living organisms (in any event their progeny will survive on Earth for a time much longer than animals and plants will do).

#### Emergence of DNA and chromosomes

In the proposed scenario, the correspondence between proteins and RNA has been established, on a one-to-one basis. RNA templates specifying proteins, together with catalytic RNAs and transfer RNAs are also replicated by RNA replicases in an RNA-genome compartment. The machinery is still quite inaccurate and rapidly exploring a variety of sequence variants. The coordination between synthesis of internal (cytoplasmic) and membrane proteins is maintained by a network of membranes filling the cytoplasm of the cell. This is (at geological time scales) a rapidly evolving situation, fairly unstable because of the lability of the ribose moiety of nucleotides. This opened up the possibility of a selectively favourable metabolic pathway where ribose would be replaced by a much stabler counterpart, namely deoxyribose. This pathway, which is unlikely to be catalysed by ribozymes, is today performed by a family of enzymes, ribonucleotide reductases [70], followed by synthesis of the corresponding nucleic acid, DNA.

The emergence of deoxyribonucleotides extended the range of cell evolution with several new functions. In particular, RNA replicases had to evolve into two activities, DNA replicases and DNA-dependent RNA polymerases (transcription), because translation was RNA-based. This makes it likely that a process resulting in the concatenation of genes developed at the same time. Indeed, the correspondence of one nucleic acid gene with one protein introduced a competition between genes. This was unlikely to sustain stable reproduction of the cells because of an inevitable quantitative mismatch between the different wielding activities of the proteins (and RNAs). Resolving this issue required some regulation allowing for their concerted transcription. A strong selection pressure that allowed for the concomitant presence of genes in a cell led to fuse them together, forming primitive multigenic chromosomes. However, this resulted in a need for identification of gene starts (promoters) as well as of control elements. Located in the promoter region these elements did not need to be transcribed, although they were replicated. The simplest way to account for their emergence is that they evolved from a combinatorial assortment of sequences of a common origin, allowing for the recognition by transcription factors that evolved in parallel. The consequence was that primitive chromosomes contained elements that were approximately repeated, thus allowing for the combinatorial association of transcription factors upstream of genes. This is the situation still witnessed in extant chromosomes of eukaryotes, but generally not in prokaryotes.

While it is important to ensure the propagation of a consistent set of genes, despite their likely huge difference in requirement as effectors of the cell metabolism, the formation of chromosomes required that the DNA replication is asymmetric, continuous on the leading strand and discontinuous on the complementary strand. It also required a machinery priming replication. Extant DNA polymerases still keep the memory of the fact that RNA preceded DNA in the initiation of replication as it remains triggered by RNA primers. The lack of homology of some of DNA polymerase constituents in the different domains of life suggests that their origin is fuzzy, with concomitant processes operating first simultaneously before the emergence of different lineages of species [71].

Like many chemical processes, replication is error-prone. This tends to produce a considerable number of mutations, leading to inactive products and sometimes “hopeful monsters” [72]. The lack of intrinsic accuracy led to proofreading and repair systems. Proofreading was ensured by the reversibility of strand elongation in the presence of pyrophosphate (and metabolic compartmentalisation of pyrophosphatase) and 3′-5′ exonuclease activity associated to the polymerases. There was also proofreading against the necessarily widespread accidental input of abundant ribonucleotides into DNA, as well as a need for mismatch repair [73]. The latter process required that the parent strand could be told from the daughter strand. As luck would have it, cytosine is unstable, as it tautomerises easily and is subsequently deaminated into uracil. This functional pressure resulted in the discovery of thymine, a DNA-specific, isosteric analog of uracil (discovered at least twice [74]). Indeed, uracil DNA glycosylase would take care of cytosine-related mutations, while the presence of uracil during replication would be used as a marker of the newly synthesised strand (when dUTP was used instead of dTTP) allowing the proofreading machinery to identify the correct strand when enabling mismatch repair.

Finally, the linear chromosomes must be synthesised in parallel with general metabolism, which developed in a 3D structure. This results in the need to make them longer than required by their strict protein-coding capacity. Another way out appears to have been via limitation of the availability of their nucleotide building blocks. Indeed, in all organisms that make de novo DNA synthesis, deoxyribonucleotides are synthesised using NDPs, not NTPs [75]. Because the concentration of NDPs is 10–100 times lower than that of NTPs the overall rate of DNA synthesis is maintained at a considerably lower level than most cytoplasmic components.

#### Escaping phagocytosis

The first cells must have associated together the progeny of an RNA-metabolism world (the ancestor of the cytoplasm with its internal membrane network) and an RNA-genome world (the ancestor of the nucleus). These protokaryotic cells explored the environment by developing engulfment processes. I have discussed elsewhere the consequence of phagocytosis: It immediately created a complementary function, that of evading phagocytosis [76]. This could be performed by at least two means, the formation of a complex engulfement-resistant envelope, or the formation of a proteolipidic cell membrane unable to fuse with that of the phagocyte. The former led to Bacteria, while the latter led to Archaea, with their membranes based on *sn*-glycerol-1-phosphate in the place of *sn*-glycerol-3-phosphate [33]. Bilayers made of mixtures of these molecules can form and are stable [77], but this is far from enough to permit functional proteolipid membrane fusion. To be sure, proteins embedded in lipid bilayers interact specifically with them [78], which constrains their ability to recognise other structures (asymmetry imposes mirror convergent evolution, see for an example the evolution of methionine sulfoxide reductases [79]). The consequence is that Archaea have envelope structures that drastically differ from those of Bacteria, and this is likely the reason for their lack of pathogenicity [80]. These escape routes allowed cells to begin to evolve toward miniaturisation, further evolving the process that had led to the formation of chromosomes, now grouping together genes with common functional associations and co-transcribing them together as operons [81]. These processes would be reflected in a stepwise evolution of the structure of proteins, as indeed observed by Caetano-Anollés and co-workers [82].

In parallel, the genome length got streamlined (remember again that DNA is linear, while size reduction goes with the third power of overall breadth), leading to a considerably dominant proportion of protein-coding genes. Furthermore, regulatory regions that were contiguous started overlapping, resulting in a progressive decrease of repeated regions. This is consistent with a common observation of genome sequences: At first sight, the genomes of eukaryotes look repeated (and therefore more primitive, with low algorithmic complexity [4]) whereas those of prokaryotes (Bacteria and Archaea alike) look random (with ”hidden” algorithmic complexity). This was however at a cost: Superposition of control regions misses the rich combinatorial possibilities of contiguous control regions, which could be used to make multicellular organisms. Modern eukaryotes came to being when protokaryotes finally succeeded in engulfing some miniaturised bacteria that were later kept as symbionts, evolving into present day organelles (mitochondria and chloroplasts). This process is still ongoing, and visible in widespread symbionts co-evolving with a large number of organisms, often multicellular [83]. While eukaryotes maintained their somewhat repetitive control regions, using them to drive the fate of a rich dictionary of differentiated cells, prokaryotes (Bacteria and Archaea) could only display a very limited range of cell differentiation, remaining essentially unicellular while retaining a still very rich family of shapes [69].

## Conclusion

At this point we have a scenario of the origin of the first cells. Admittedly, it is somewhat heterodox (many still consider bacteria as “primitive”), but consistent with what we know about the evolution of biological (and engineering) functions. This scenario is based on the accumulation of specific functional constraints, beginning with selection by surfaces of a subset of charged chemical compounds that react together, creating building blocks for future macromolecules, as well as coenzymes essential for catalysis. Among these molecules are the first nucleotides, which begin to polymerise, substitute charged surfaces as substrates for metabolism then, via nucleic-base complementarity, explore the role of template for coded peptide synthesis. In parallel, a rich network of membrane structures is emerging, allowing the cell to create electrochemical gradients that are used to drive exchanges between the cell and the environment, as well as processes of growth, fusion, fission and engulfement (Figure 1). After emergence of RNA replication, cells evolve via combining an RNA-metabolism world and an RNA-genome world [25,76].

Rapidly, these cells are stable enough to survive for a significant amount of time. This is enough to create a new challenge, that of ageing. Indeed all metabolites and macromolecules will change over time, simply because of their spatial and physico-chemical constitution. We have already observed a consequence of this inevitable burden in the recruitment of deoxyribose and thymine, leading to DNA as a memory, compensating for instability of ribose and cytosine. In proteins, ageing is manifest in the ubiquitous spontaneous cyclisation of aspartate and asparagine residues [84]. The consequence is that cells progressively become bags of products of different age. In general (but not always, as the positive consequences of time-dependent maturation tells us) aged compounds will lack proper functional capacities. The cell will progressively become senescent and then die.

A way out is to create a progeny. However it is essential that this progeny is chiefly composed of young compounds. This creates a remarkable challenge: How can the cell keep old compounds in the parent, while the daughter cell will essentially be composed of young compounds? This question is reminiscent of the question tackled by James Clerk Maxwell when discussing his “Theory of Heat” [85]. How could we separate moving gas molecules according to their speed, if we could have an enclosure split into two compartments by a thin wall with an opening trap that could be opened or closed at will? Maxwell proposed that an intelligent being (later named Maxwell's demon) could measure the speed of incoming molecules and either open or close the trap, according to their measured speed. This process retained all fast molecules on one side (making it hot) and the slow one on the other side (making it cold). If this were possible, this would allow one to create a steam machine, and hence a perpetual movement, as it appeared that it could be possible to use such a demon without energy. This was discussed for decades until a fairly final demonstration by Rolf Landauer followed by Charles Bennett showed that acquiring memory (computing) indeed does not require energy, but that erasing memory will, so that the process is indeed energy consuming, precluding perpetual movement [86–87]. Apart from the trap mechanics, many other processes would settle the conundrum. Besides separating things according to their age into two compartments, another way would be, for example, to evolve specific devices (other types of Maxwell's demons) that patrol within the cell compartment, consistently interacting with molecules there (via a selective process of complementarity), and destroying those that have aged, then using ATP or GTP hydrolysis to reset their memory for another fruitful interaction. This latter way of coping with altered components of the cell has been shown to be consistent with the law that illustrates the probability of death in most living organisms, Gompertz law [88].

This requirement, making a young progeny, asks that cells provide the code for objects, likely proteins, operating as Maxwell's demons [89]. Notably, if living organisms code for Maxwell's demons that select and maintain cells in a way that accumulates information, these demons have highly specific families of targets, or are located spatially at precise sites. They cannot have any global grand design. Because these demons are only local they cannot directly organise the whole of a multicellular organism in a single step. This may explain why many organisms undergo metamorphoses, with specific stages, each one essential to promote the smooth unfolding of the next stage. This also explains why the final outcome of their activity is akin to tinkering, as François Jacob put it (making “kludges” might be a more appropriate word), and leads to the extraordinary diversity of life forms (that often look gratuitous). In some situations, however, physical constraints may restore some order in the outcome (for example spheres, tubes, the pythagorean/platonic regular polyedra of viral capsids, and more complex structures, such as phyllotaxis, the way leaves are distributed along a stem, or flowers within a composite inflorescence [90]). Yet these constraints, contrary to the great expectations of laypersons looking for evidence of design in living organisms, do not say much about what life is. They just provide borders within which information-rich physical systems (information gathering and utilising systems as named by Gell-Mann [91]) can explore reality.

The central feature of what is life is that of a specific way to manage information. The main problem with this general function is that it must be performed via a material set-up, putting together matter and energy in order to manipulate information. The consequence is that we must consider several quite different levels of description. There is a completely abstract level, that only considers the fate of information (this is the idea of Maxwell's demons in biology), and there is a series of more concrete levels that involve the machine, with its idiosyncrasies, that reads the genetic program. The latter involve the necessary constraints operating on matter and its coupling to energy in the set-up of life as we know it on Earth. This is where engineering has to be called for. All this is fairly similar to what happens when engineers construct computers. At the abstract level we have the Turing machine, so abstract that nothing is said about the innards of the machine. We have physical constraints operating on the global behaviour of the machine (management of heat in particular) and we have the many kludges of the explicit manifestation of a personal computer.

The main question we have to tackle, then, is to articulate the way we link a conceptual view of what life is, to experiments meant to make it in concrete terms. A large fraction of the design of what is a cell is now understood. This is what came out in the *Mycoplasma mycoides* JCVI Syn3.0 construct [92], after one has gone further than the original paper, with identification of much of the “unknown” functions [93]. There, we find a set-up of the Turing machine, with a concrete implementation of the reading heads of the program, the ribosomes. While the concept is well understood, there is not much latitude to modify what has been selected during the 3.5 billion years of evolution. It seems difficult, if not plainly impossible, to “re-invent” a ribosome, but we can study variations upon this theme. The same is true for replication and for a first level of cell division. However there remains an enigma that is amenable to experiments. How, in these constructs, is a young progeny created? It would be extremely interesting to see how the colonies formed with the JCVI Syn3.0 construct can be reproduced over many generations, perhaps by streaking them on plates of constant composition, to see whether this is at all possible (i.e., see whether or not there is a finite number of possible generations) and, if so, to see how the system evolved. We should remember that the first example of a similar construct, with a much smaller genome, and not of a living cell but of a bacteriophage (T7) evolved via erasing at least one third of the human construct [94].

If this experiment does not result in a progressive degeneracy of the genome, as doomed to happen if Muller's ratchet operates and drives an error catastrophe [95–96], exploring the genome after many generation will allow us to decipher how key functions in a minimal genome could lead to emerging novel functions. Functional analysis tells us that there is always an open door to a novel function [97], provided an existing structure is promiscous enough to allow that function to operate. Losing a function such as the protease that is required to maturate protein L27 in the ribosome of the Syn3.0 construct [93] might well recruit another endopeptidase for that particular function, for example. However, with this streamlined genome there is not much room left to trap the contextual information related to the process of evolution. This is exactly where gene duplication may come in [98], knowing in particular that selection pressure tends to increase the length of the chromosome to match the three-dimensional metabolism of the cytoplasm, as we have seen. A way out would be a spontaneous duplication of some or all of the genome, creating room for innovation. This apparently neutral process would in fact create novel information by allowing the cell to create a novel asymmetry, typical of what is needed for creation of information. We can suspect that a large number of sequences interpreted by many authors as “useless” in genomes [99] are in fact a way for those to prepare for the future.
